# Refractory versus resistant invasive aspergillosis

**DOI:** 10.1093/jac/dkaf003

**Published:** 2025-03-14

**Authors:** Maiken Cavling Arendrup, Catherine Cordonnier

**Affiliations:** Unit of Mycology, Statens Serum Institut, Building 45, room 123, Artillerivej 5, DK-2300 Copenhagen, Denmark; Department of Clinical Microbiology, Rigshospitalet, Copenhagen, Denmark; Department of Haematology, Henri Mondor Teaching Hospital, Assistance Publique-Hôpitaux de Paris, and Université Paris-Est-Créteil, Créteil, France

## Abstract

Despite notable progress, the management of invasive aspergillosis (IA) remains challenging and treatment failures are common. The final patient outcome is subject to multiple factors including the host (the severity of the underlying conditions), the fungus (the virulence and susceptibility pattern of the *Aspergillus* species involved), and the therapy (the timing related to severity of infection and choice of therapy—dose, efficacy, cidal versus static, toxicity and interaction). Consequently, assessment of failure is complex yet crucial in order to ensure appropriate management.

Refractoriness in absence of drug resistance may reflect severity of the underlying disease/infection at the time of initiation of therapy prolonging time to response. It may also reflect a suboptimal antifungal drug exposure due to poor compliance, inappropriate dosing or increased drug metabolism, or it may reflect ‘pseudo’ failure due to worsening of imaging due to recovery of neutrophils. Refractoriness may also be related to inherent drug resistance in various *Aspergillus* species or acquired resistance in a normally susceptible species. The latter scenario is mostly encountered in *A. fumigatus*, where azole resistance is increasing and includes azole-naive patients due to resistance related to azole fungicide use in agriculture and horticulture. Although diagnostics and resistance detection have been greatly improved, the time to resistance reporting is often still suboptimal, which calls for close assessment and potentially management changes even before the susceptibility is known.

In this article we address the various definitions and approaches to assessment and management of clinical refractoriness/failure in the setting of proven and probable IA.

## Introduction

Although considerable progress has been made over the last decades in the treatment of invasive aspergillosis (IA), the disease remains life-threatening in the most immunocompromised patients. With definitions evolving according to the available diagnostic methods and guidelines for assessing the antifungal response in prospective trials,^1,2^ the quality of the studies has considerably improved, allowing several groups to propose evidence-based recommendations for routine practice.^3–5^ However, a significant number of patients still fail to respond.^6–11^ The criteria for failure used in prospective trials were not designed for routine practice. Moreover, clinicians and mycologists in some areas face increasing resistance to triazoles, adding to the many causes of refractoriness and even failure.

In this article we discuss the assessment of failure in IA, the various approaches hereto proposed in the literature, and highlight the need for a complete investigative work-up of these situations, at least when the status of the underlying disease allows hope for a cure or stabilization of IA. We limit our review to proven and probable IA, excluding possible cases where the certainty of a true infection is vague. We will also not address intolerance or drug–drug interactions as causes for switching to another class of antifungals, nor the different choices of antifungals for salvage treatment, a topic that is widely addressed in guidelines.^3–5^

## Refractoriness and resistance are not the same

### Main concerns for assessing the response of IA to antifungals

The therapeutic response of mould infections is known to be slow, and persisting abnormalities may be visible on imaging years after the clinical cure, especially in lungs.^12,13^ The clinical signs and the general status of the patient may be difficult to interpret in these patients threatened by multiple events, new infections or drug toxicities. These patients also often suffer from mixed infections, jeopardizing the assessment of antifungal activity. The interpretation of imaging evolution under treatment often needs expertise and a close collaboration with the clinician since the increase in size of CT lesions is expected at neutrophil recovery, even in favourable outcomes.^14^ For microbiological assessment, resampling the initial lesions may be difficult, especially in lungs or the CNS. Therefore, most information relies on serum galactomannan guided response, which implies an initial positive and quantifiable level and the lack of confounding factors (e.g. renal failure, neutrophil recovery) complicating its interpretation.^15^ Finally, the evolution of these clinical, radiological and mycological criteria may be discordant or not strictly concomitant, especially if assessed early on.

### Definitions of refractoriness in the literature

Although refractoriness is often not defined as such in prospective trials, we may consider that it should be close to the definitions of failure—death excluded—in first-line treatment trials and to the inclusion criteria of second-line/salvage trials. First-line trials have mostly defined success as the sum of complete (rarely obtained) and partial response, and failure as the sum of stability and of worsening of symptoms, including any situation where the reduction in the size of images was ≤50%, assessed at end of therapy when prematurely stopped, or at 6 or 12 weeks.^6–10^ Adopting these criteria, the rate of failure was between 35% and 66%. The way the images were measured is not always known or comparable from one study to the other. Indeed, investigators used alternatively the diameter^8^ as recommended by Segal *et al*.,^2^ the area,^10,12^ or an estimated volume of the lesions.^16^

Although survival was not considered as a sufficient criterion of antifungal response by the European Organization for Research and Treatment of Cancer Mycoses Study Group (EORTC-MSG) expert panel in 2008,^2^ overall survival was used as the primary endpoint in the most recent trials of first-line treatment.^7–9^ This choice may be explained by the binary information provided by survival (although the cause of death is rarely documented) and the fact that none of the previous criteria (e.g. decrease in the size of the lesions, rarity of complete cure) was satisfying.

Second-line/salvage trials mostly included patients either intolerant to a previous antifungal (12%–14%) or with stable or progressive IA despite a minimum of 7 days of first-line treatment. Stable disease was again defined by a <50% reduction of the lesions.^6–10^ It should be noted that resistance data were rarely available in these trials.

### The many causes of refractoriness

As there is no clear indication in the literature to know when to stop antifungals, there is no clear indication regarding when to switch to a second-line treatment, both being likely dependent on the immune status of the patient.^13,17^ Large first-line, prospective trials showed that the duration of the study-drug first-line treatment was very variable, between 15^10^ and 77 days.^6^ This duration may have been influenced by the planned duration of the antifungal treatment in the study protocol and by the route of administration. The main causes of refractoriness are listed in Table 1, resistance to the administered antifungal being one of them. It should be noted that among these causes, the most difficult to investigate is the deepness of immunosuppression, especially regarding fungi, given the lack of routine immunological tests of fungal immunity. All prospective trials used consensus criteria for defining proven, probable and possible infections, primarily adhering to the successive versions of the EORTC-MSG criteria. These criteria outline the most common immunocompromised conditions at risk of invasive fungal disease, including recent history of neutropenia, allogeneic stem cell transplantation, prolonged use of corticosteroids, and inherited severe immunodeficiency. However, these trials did not provide additional precision in assessing the baseline severity or the outcomes of immunosuppression during treatment.

**Table 1.** Main causes and confounding factors of refractory invasive aspergillosis

**Host factors**
Poor compliance, poor absorption, drug–drug interactions
Dose-limiting toxicity
Profound immune depression
Altered drug disposition or metabolism (genetic inheritance regarding drug metabolism, overweight, organ failure, dialysis, extracorporeal membrane oxygenation)
Lung tissue status and architecture
**Therapy-related factors**
Delay in treatment initiation
Suboptimal doses of antifungal(s) compared with MIC
Suboptimal antifungal concentration at site of infection (CNS, intra-abdominally, sinuses)
**Fungal factors**
Initial high fungal burden
Biofilm formation
Virulence
Growth kinetics
Natural or acquired resistance
**Confounding factors**
Errors in the initial diagnosis
Initial mixed susceptible and resistant *A. fumigatus*
Initial coinfection(s) (including with other fungi or in the IA site)
Other infections occurring during the follow-up of IA

## Refractory invasive aspergillosis

Although different from one to the other, the guidelines or proposals for defining refractory IA all agree on the need to assess the treatment response with a composite set of clinical, radiological and mycological criteria.

### Main available guidelines for managing refractory IA

In the guidelines proposed by Segal *et al*.^2^ for clinical trials, failure included stability (<25% reduction in the diameter of the lesions) and progression as a clinical worsening, the occurrence of new lesions, increase of the initial lesion(s) or persistent isolation of the mould from infected sites. Although discussed, the use of galactomannan for assessing the treatment response was not adopted by the expert panel in 2008 although it was later recognized as a useful marker of response and outcome in the 2016 IDSA guidelines.^3^ However, no international guidelines give useful guidance for switching to a second-line agent in routine practice. In the absence of documented resistance, both the ESCMID and the American Society of Transplantation and Cellular Therapy (ASTCT)^5,18^ proposed a first assessment after Day 14 of treatment, considering that increase in size of the initial lesion is expected over the first 2 weeks in responding cases and earlier assessment therefore would be premature.^12–14^ As for biomarkers, we agree that galactomannan is until now the only reliable marker for assessing the therapeutic response of IA, although it may be produced by other fungi.^19^ This issue has been extensively reviewed by Mercier *et al*.,^15^ showing that galactomannan is a reliable and early marker of outcome in IA from Day 7, although no cut-off of response is established.

Recently, a group of experts has proposed a list of reasons for changing first-line antifungal treatment in IA earlier than previously suggested in routine practice (Table 2).^20^ These included detection of resistance at any timepoint, and otherwise to introduce a first assessment from Day 8 of treatment taking into account increasing galactomannan indices, onset of new lesions and clinical deterioration, but postponing the assessment of the size of the initial lesions from Day 15. Although not yet validated, these proposals provide realistic guidance to the clinician, based on available routine diagnostic methods.

**Table 2.** Reasons proposed by Slavin *et al.*^20^ for changing first-line antifungal treatment

Days since initiation of therapy	Clinical and diagnostic findings compared with baseline
At any time	Identification of a pathogen resistant to primary antifungal therapy
8 to 14 days	On the basis of changes in GM:
	1. Serum: The serum GM index has not fallen by 1 unit or become negative (GM index <0.5) based on measurements taken at least 7 days apart
	1. BAL: Positive GM from BAL in a patient with a previous BAL test that did not meet the definition of positive (too low or entirely negative) without regard for the interval of time between samples. Note that there is no definition for rising GM index values from BAL as these values are subject to sampling error
	*Or*
	Clinical deterioration consistent with persisting or progressive invasive fungal disease with no other identifiable aetiology
	*Or*
	New distinct site of infection detected clinically or radiologically
≥15 days	Any of the above criteria
	*Or*
	Progression of original lesions on CT (or other imaging) based on >25% growth of initial lesions in the context of no change in immune status

BAL, bronchoalveolar lavage; GM, galactomannan antigen.

Breakthrough infections (mostly occurring more than 3–7 days after starting prophylaxis) were also addressed in the literature, especially focusing on the need to check adequate drug levels during prophylaxis and to document the IA before switching.^20,21^

## Resistant IA

### Inherent and acquired resistance

Resistant aspergillosis involves two categories: (i) infections with *Aspergillus* species that possess inherent (also known as intrinsic) resistance; and (ii) infections with *Aspergillus* isolates with acquired resistance. *Aspergillus* includes (at least) 148 species belonging to 23 sections, 14 of which include at least 58 species that have been associated with human disease.^22,23^ A number of these display resistance or reduced susceptibility to one or several of the three drug classes currently available for medical therapy (Table 3). In these cases, correct species identification is informative for selecting therapy, e.g. avoiding amphotericin B for *A. terreus* and *A. flavus* infections, and avoiding azoles for infections due to *A. calidoustus*. However, a robust correlation between appropriate therapy according to MIC for cryptic species and outcome is not always found. This is likely because these species are more difficult to diagnose, resulting in more delayed therapies, and are presumably less virulent given the fact they rarely cause disease, which means that the outcome is more affected by the severity of the underlying host disease than by the infection itself compared with infections due to the more virulent *A. fumigatus*.^24^

**Table 3.** Overview of susceptibility pattern of the common and selected rare/cryptic species compared with that of WT *A. fumigatus*^a^

Species	Isolates (*n*)^b^	Amphotericin B	Itraconazole	Posaconazole	Voriconazole	Isavuconazole	Olorofim
**Common species**							
*A. fumigatus*	(>1400)	+	+	+	+	+	+
*A. flavus*	(207; 48)	(+)	+	+	(+)	+	+
*A. terreus*	(188; 64)	—	+	+	(+)	+	+
*A. niger*	(185;129)	+	(+)	+	(+)	(+)	+
*A. nidulans*	(121; 17)	(+)	+	+	+	+	+
**Cryptic species**							
*A. hiratsukae*	(12; 2)	+	+	+	+	+	+
*A. latus*	(30)	+	+	+	+	+	+
*A. montevidensis*	(3)	+	+	+	+	+	—
*A. thermomutatus*	(18; 16)	+	—	—	—	—	+
*A. felis*	(4; 2)	(+)	—	(+)	—	(+)	+
*A. fischeri*	(3)	(+)	—	(+)	(+)	(+)	ND
*A. calidoustus*	(18; 8)	(+)	—	—	—	—	ND
*A. alliaceus*	(12)	—	+	+	+	+	+
*A. westerdijkiae*	(4)	—	+	(+)	+	+	+
*A. sydowii*	(34; 55; 12)	—	(+)	(+)	(+)	(+)	+
*A. lentulus*	(9; 2)	—	(+)	(+)	—	(+)	ND
*A. udagawae*	(4)	—	—	(+)	—	(+)	ND

ND, no data found.

^a^+ Indicates MICs comparable to those of *A. fumigatus* (similar or lower modal MICs and ranges); (+) indicates MIC 1–2 dilutions higher than that for *A. fumigatus*; — indicates MIC  ≥4 dilutions higher than those against *A. fumigatus.*

^b^MIC data from EUCAST: rationale documents for antifungals retrieved 12 December 2023 and supplemented by data from references ^24–26^. Number of isolates from individual sources is indicated and separated by a semicolon. For the species with few isolates in the included publications (*A. montevidensis, A. fischeri*, *A. westerdijkiae* and *A. udagawae*) the pattern aligns with additional unpublished in-house MICs; however, more data are warranted for these species.

### Epidemiology of azole-resistant A. fumigatus

Acquired azole resistance in *A. fumigatus* has become an important cause of resistant IA and refractory disease, which in some regions or settings challenges the use of azoles as initial therapy of IA.^27,28^ Azole-resistant IA can arise after long-term azole use in the individual patient where awareness of the prior azole therapy may prompt suspicion of resistance development.^29–33^ However, azole-resistant *A. fumigatus* has also emerged in the environment as a consequence of widespread use of azole fungicides to control fungal plant disease and therefore cause azole-resistant infection in the azole-naive host. *A. fumigatus* is not a plant pathogen and not the target of environmental fungicide use, yet primary infections due to inhalation of resistant *A. fumigatus* from the environment have become increasingly important since the first description in 2007 in an azole-naive patient.^34^ Today, azole-resistant *A. fumigatus* has been reported in the environment in multiple settings and countries worldwide, although at different rates,^27,35,36^ and accounts for approximately half of the azole-resistant cases in a recent nationwide survey in Denmark of *A. fumigatus* in clinical samples.^37^ The majority of environmental azole-resistant *A. fumigatus* isolates harbour a tandem repeat (TR) in the promoter region of the target gene resulting in up-regulation of its expression, combined with point mutation(s) in the target gene *cyp51A* compromising the affinity of azole to the CYP51A enzyme. The most common variants are TR_34_/L98H (pan azole resistant) and TR_46_/Y121F/T289A (highly resistant to voriconazole and isavuconazole), but variants hereof continue to emerge.^38,39^

### Epidemiology of azole-resistant A. flavus and A. terreus

Acquired azole resistance in *A. flavus* and *A. terreus* is less studied and likely underreported, as susceptibility testing is not universally performed. Mutations have been found in *cyp51A*, *cyp51B* and *cyp51C*^40–46^ of azole-resistant *A. flavus* isolates but only a few have been confirmed as causes of resistance. Efflux pump activity has also been found and these mechanisms may play in concert.^40,47^ Acquired azole resistance in *A. terreus* has been found in isolates with mutations in *cyp51A* from azole-treated patients, but it is uncommon and was not found in environmental samples in a recent study.^33,48,49^

### The clinical impact of azole resistance

The clinical impact of rising azole resistance in *A. fumigatus* is significant. Resistance often comes as a surprise particularly in the azole-naive patient. In a recent multicentre study^28^ it delayed appropriate therapy and caused significantly poorer outcomes, with survival rates decreasing from 72% to 51% at Day 42, and from 63% to 38% at Day 90, when comparing patients with IA due to susceptible versus resistant *A. fumigatus*. Of note, appropriate change of therapy from voriconazole to amphotericin B when susceptibility testing identified voriconazole resistance did not prevent a lower survival compared with patients with a susceptible isolate (53% versus 76% survival).^28^

### Management of IA in the setting of azole resistance

The increasing azole resistance rates call for an evaluation of the management of IA. Although a first thought might be simply to change initial therapy to amphotericin B, particularly in countries with a high incidence of resistance, this may not be the optimal solution, given that the efficacy against susceptible *A. fumigatus* (which still forms the majority) varies between the drug classes. Based on available clinical trials, the success rates against susceptible *A. fumigatus* are 70% for voriconazole, 60%–65% for liposomal (L) amphotericin B and 35% for echinocandins.^6,10,50–56^ Consequently, even if assuming no efficacy at all for voriconazole against resistant *A. fumigatus*, the estimated overall success rate for voriconazole monotherapy will be superior or equal to that for L-amphotericin B (success rates of 65%–60%) for voriconazole resistance rates below or equal to 7%–14%. Similarly, estimated voriconazole-echinocandin combination therapy will be superior or equal to that for L-amphotericin B for azole resistance rates of 14%–28%. Based on these estimates, an international expert opinion panel provided guidance on the management of IA in regions with low versus high (>10%) resistance rates in clinical practice.^57^ In countries with a low azole resistance rate, voriconazole is recommended as the first-line agent for IA, but emphasis is given on rapid MIC (within 72 h) in regions where azole resistance has been detected. In countries with high resistance rates, initial therapy with either a voriconazole/echinocandin combination or with L-amphotericin B is recommended. Also, to adjust therapy to amphotericin B in case resistance is verified, either through susceptibility testing or molecular detection of resistance mutations, or to voriconazole monotherapy if resistance is not confirmed. However, in a number of cases, diagnosis is established without culturing *A. fumigatus*, and even when molecular detection of resistance is available, it may detect resistance but cannot detect susceptibility given the lower sensitivity for the single-copy target gene compared with the multicopy gene used for *A. fumigatus* detection. Moreover, a considerable proportion of infections include both susceptible and resistant phenotypes.^37^ In these cases combination therapy should be continued for at least 2 weeks before careful de-escalation with close monitoring (galactomannan, PCR, culture, imaging and azole concentration determinations).^57^

## Why it is important to investigate the suspicions of failures

As the causes of refractoriness are many (Table 1), an extensive diagnostic work-up is crucial to understand the failure in individual cases and take the appropriate measures, especially when the status of the underlying disease allows a cure or at least a durable remission.

### Failures/lack of response despite apparently appropriate drug choice

In the absence of documented resistance, the initial choice should be considered appropriate as far as it is consistent with international guidelines. However, it should be noted that the lack of detection of resistance does not equal susceptibility, because coinfection with resistant isolates may not be represented in the investigated sample, or new resistance mechanisms continue to arise, which may not be covered by molecular detection assays.^37,39^ Therapeutic azole monitoring should eliminate suboptimal exposure to the drug related to host or dosing factors (Table 1). All patients with suspected treatment failure after at least 7 days of first-line therapy should undergo further investigation of the initial site to reassess the diagnosis and exclude possible coinfection by a resistant fungus not initially identified. This may include repeat bronchoalveolar lavage and/or CT-guided biopsies.^20^ With a confirmed lack of azole resistance, the choice should be adapting the dose rather than changing the antifungal class.

### Failures/lack of response due to resistance: diagnostic and treatment implications

In case of resistance, the treatment should be changed as quickly as possible as treatment delay may worsen outcome. This was shown in several studies in the setting of candidaemia,^58,59^ but it is also shown by the worsened outcome in azole-resistant IA despite change of initial azole to L-amphotericin B.^28^

## Current achievements and future needs

The rising incidence of azole resistance has complicated management and raised issues that need to be addressed. Establishment of surveillance programmes to inform on local epidemiology is crucial and necessary to ensure appropriate initial therapy according to local resistance rates. This has recently been acknowledged by the WHO, which introduced a pivotal fungal priority pathogens list in late 2022; this includes *A. fumigatus* but still only a few countries have systematic surveillance programmes for *Aspergillus*.

Many questions remain to be solved (Table 4). A special consideration should be given to fluorodeoxyglucose (FDG) positron emission tomography (PET)/CT imaging. The superiority of PET over classical CT has been mainly illustrated in the long-term follow-up of IA to identify the active lesions that do not disappear on the CT. But its role and potential advantages specifically in the early weeks of infection remain to be evaluated. Moreover, the use of fungal-specific radiotracers appears to be promising for this purpose.^60,61^

**Table 4.** Main unsolved questions for assessing the response of IA to antifungal treatment and switch of therapy in routine practice

Topics	Questions
Clinical	Should a stable disease in an immunocompromised patient whose level of immunosuppression does not improve be considered as a reason to change the antifungals?
Biomarkers	Can a cut-off of response be established for serum galactomannan when initially positive?
	Can β-D-glucan or quantitative PCR in serum and BAL fluid be useful tools of follow-up?
	Are there other reliable biomarkers of IA to help?
Imaging	Can PET-CT scan be useful for the early assessment of IA, especially if combined with fungal-specific biomarkers?
Antifungals	Does a fungicidal versus a fungistatic effect impact the kinetics of the response differentially?
	Do the mechanism and kinetics of action of new antifungals influence the timing of the response? Can combinations of two antifungal drug classes either: –improve efficacy, or –reduce side effects by allowing lower doses?

BAL, bronchoalveolar lavage; PET, positron emission tomography.

Perhaps equally important as improved laboratory diagnostics is timeliness of diagnostics, with a rapid turnover time and reports in real time. Classic diagnostics with a focus on the use of optical brighteners and high-volume cultures on selective agars, use of azole screening agars and validated susceptibility testing remain crucial.^62,63^ Identification to the species level by MALDI-TOF is now a reliable tool to identify *Aspergillus* at the section level provided the appropriate database is used.^64^ This facilitates rapid species identification and adjustment of therapy for cases involving species with inherent resistance. Biomarkers specific for *A. fumigatus* include antigen testing and PCR, which further advance our access to rapid diagnostics. Galactomannan detection is commercially available as an ELISA version for batched testing and in formats convenient for frequent testing of lower number of samples.^65,66^ Similarly, commercial PCR assays specific for *Aspergillus* that include species identification and detection of the two most common environmental azole resistance mechanisms are available.^67^

But despite these achievements in diagnostics and faster identification of resistant cases, new antifungals with efficacy against azole-resistant *A. fumigatus*, available in IV and oral formulations and with agreeable toxicity and drug–drug interaction profiles, are highly warranted. In this context, it is promising that two new drug classes, the dihydroorotate dehydrogenase (DHODH) inhibitor olorofim and the Gwt1 (glycosylphosphatidylinositol-anchored wall protein transfer 1, which catalyses the third step of the glycosylphosphatidylinositol anchor biosynthesis pathway) inhibitor fosmanogepix, are currently being investigated in prospective trials (olorofim NCT05101187 and NCT03583164; fosmanogepix NCT04240886; https://www.clinicaltrials.gov). However, it is frustrating that agents belonging to the same drug classes are in development for use in agriculture: ipflufenoquin and quinofumelin (both targeting DHODH) and aminopyrifen (targeting Gwt1).^68^ Olorofim resistance in *A. fumigatus* has been selected *in vitro*,^69^ and a correlation between olorofim and ipflufenoquin susceptibility demonstrated against *Aspergillus* species.^68^ These observations suggest a risk of selecting olorofim/manogepix resistance in *Aspergillus* if fungicides of the same drug classes are used as fungicides in the environment.

In conclusion, significant advances have been made in recent years in our understanding, diagnosis and management of aspergillosis, as well as in addressing refractoriness and treatment failure. Furthermore, new therapeutic options are in development and may provide viable alternatives for patients who are intolerant to current agents or have refractory or resistant infections. These achievements, if antifungal stewardship measures are adhered to in both clinical medicine and agriculture, hold the potential to improve outcomes for patients with proven or probable IA in the future.

## Transparency declarations

The authors declare the following potential conflicts of interest outside this work: M.C.A. has over the past 5 years received research grants/contract work (paid to the Statens Serum Institut) from Cidara/Mundipharma, F2G/Shionogi and Scynexis, and speaker’s honoraria (personal fees) from Gilead and F2G/Shionogi. She is the current chairman of the EUCAST-antifungal susceptibility testing subcommittee (AFST). C.C. has over the past 5 years received personal speaker’s honoraria from F2G/Shionogi, Gilead Science, and Merck Sharp & Dohme, and consulting fees from Cidara, Merck Sharp & Dohme, and Mundipharma.
